# Physiological Responses to Acute Cycling With Blood Flow Restriction

**DOI:** 10.3389/fphys.2022.800155

**Published:** 2022-03-11

**Authors:** Matthew A. Kilgas, Tejin Yoon, John McDaniel, Kevin C. Phillips, Steven J. Elmer

**Affiliations:** ^1^School of Health and Human Performance, Northern Michigan University, Marquette, MI, United States; ^2^Department of Kinesiology and Integrative Physiology, Michigan Technological University, Houghton, MI, United States; ^3^Department of Physical Education, Kangwon National University, Chuncheon, South Korea; ^4^Department of Exercise Physiology, Kent State University, Kent, OH, United States; ^5^Louis Stokes Cleveland Veterans Affairs Medical Center, Cleveland, OH, United States

**Keywords:** vascular occlusion, Kaatsu, aerobic exercise, fatigue, arterial blood flow, functional near inferred spectroscopy

## Abstract

Aerobic exercise with blood flow restriction (BFR) can improve muscular function and aerobic capacity. However, the extent to which cuff pressure influences acute physiological responses to aerobic exercise with BFR is not well documented. We compared blood flow, tissue oxygenation, and neuromuscular responses to acute cycling with and without BFR. Ten participants completed four intermittent cycling (6 × 2 min) conditions: low-load cycling (LL), low-load cycling with BFR at 60% of limb occlusion pressure (BFR60), low-load cycling with BFR at 80% of limb occlusion pressure (BFR80), and high-load cycling (HL). Tissue oxygenation, cardiorespiratory, metabolic, and perceptual responses were assessed during cycling and blood flow was measured during recovery periods. Pre- to post-exercise changes in knee extensor function were also assessed. BFR60 and BFR80 reduced blood flow (~33 and ~ 50%, respectively) and tissue saturation index (~5 and ~15%, respectively) when compared to LL (all *p* < 0.05). BFR60 resulted in lower VO_2_, heart rate, ventilation, and perceived exertion compared to HL (all *p* < 0.05), whereas BFR80 resulted in similar heart rates and exertion to HL (both *p* > 0.05). BFR60 and BFR80 elicited greater pain compared to LL and HL (all *p* < 0.05). After exercise, knee extensor torque decreased by ~18 and 40% for BFR60 and BFR80, respectively (both *p* < 0.05), and was compromised mostly through peripheral mechanisms. Cycling with BFR increased metabolic stress, decreased blood flow, and impaired neuromuscular function. However, only BFR60 did so without causing very severe pain (>8 on pain intensity scale). Cycling with BFR at moderate pressure may serve as a potential alternative to traditional high-intensity aerobic exercise.

## Introduction

Exercise with blood flow restriction (BFR) offers an alternative method for increasing muscle size and strength (Nakajima et al., 2006; Loenneke et al., 2012b). This exercise uses a pressurized cuff or tourniquet to restrict blood flow to and from the working muscles (Scott et al., 2015). While BFR is usually combined with low-load resistance exercise (e.g., 20–30% of one repetition maximum; Patterson and Brandner, 2017), it has also been used with low-intensity aerobic exercise (e.g., <50% of VO_2peak_). Aerobic exercise with BFR increases muscle size and strength as well as VO_2peak_, time until exhaustion, and onset of blood lactate accumulation (Abe et al., 2010; Corvino et al., 2014; De Oliveira et al., 2016). Thus, aerobic exercise with BFR may offer a unique stimulus for improving both muscular function and aerobic capacity.

Selection of cuff pressure is critical for administering exercise with BFR safely and effectively. According to a recent BFR position stand (Patterson et al., 2019), cuff pressures between 40 and 80% of an individual’s resting limb occlusion pressure are recommended. Application of pressure within this range is thought to improve muscular and cardiovascular function by augmenting metabolite accumulation and local hypoxia (Scott et al., 2014; Cayot et al., 2016), and inducing cell swelling (Loenneke et al., 2014). It has been proposed that these metabolic changes accelerate fatigue leading to preferential type II fiber recruitment (Yasuda et al., 2009), increase serum growth hormone (Pierce et al., 2006), and stimulate angiogenesis, mitochondrial biogenesis (Conceicao et al., 2019), and increase arterial diameter (Christiansen et al., 2020). While cuff pressures are typically set as a percentage of resting limb occlusion pressure, the impact they have on the magnitude of blood flow during exercise and intermittent recovery periods is not well established. We previously assessed changes in blood flow (*via* Doppler ultrasound) and tissue oxygenation (*via* near-infrared spectroscopy) while performing rhythmic handgrip (Kilgas et al., 2019) and knee extension (Singer et al., 2020) contractions across a range of cuff pressures. Our results indicated that pressures of 60 and 80% of limb occlusion pressure decreased blood flow and tissue saturation and increased concentrations of deoxyhemoglobin. Together, these findings support the notion that moderate cuff pressures increase metabolic stress and metabolite accumulation without completely occluding blood flow and compromising individual safety. It is unclear if these changes for resistance-based exercise with BFR can be applied to aerobic exercise with BFR because of differences in blood pressure, cardiac output, muscle mass, cyclical nature of the movement, and time under occlusion between the two exercise modes.

Reductions in blood flow during BFR exercise also contribute to the development of neuromuscular fatigue (i.e., reduction in maximal voluntary torque; Bigland-Ritchie and Woods, 1984). These reductions in maximal voluntary torque could be due to factors that reside in the brain and spinal column (central fatigue), the muscles themselves (peripheral fatigue), and/or some combination of the two (central and peripheral fatigue). Typically, greater neuromuscular fatigue is associated with increased growth hormone concentrations which may play a role in training adaptations (Hakkinen and Pakarinen, 1993; Pierce et al., 2006). Previous authors (Karabulut et al., 2010; Cook et al., 2013; Husmann et al., 2018) have reported impairments in neuromuscular function following knee extension exercise with BFR which were attributed to both central and peripheral mechanisms. Changes in neuromuscular function following aerobic exercise with BFR, however, are mixed with some authors reporting reduced knee extensor torque after submaximal (Kim et al., 2015) and supramaximal (Willis et al., 2018) cycling but not after walking (Ogawa et al., 2012). Given these varied results and the task-specific nature of fatigue (Bigland-Ritchie et al., 1995), more work is needed to confirm how cuff pressure influences the development of fatigue during aerobic exercise with BFR.

To date, the extent to which cuff pressure influences acute physiological responses to aerobic exercise with BFR is not well understood. Therefore, the purpose of this study was to compare changes in blood flow, tissue oxygenation, and neuromuscular function to acute cycling exercise with and without BFR. We hypothesized that cycling with BFR (60% of limb occlusion pressure) would decrease blood flow and tissue saturation index and increase concentrations of deoxyhemoglobin. These responses would be further exacerbated by increased cuff pressure (80% of limb occlusion pressure). We also hypothesized that cycling with BFR would impair end exercise maximal knee extensor isometric torque. Based on previous reports (Karabulut et al., 2010; Cook et al., 2013; Husmann et al., 2018), we hypothesized that these impairments in neuromuscular function would be to central and peripheral mechanisms. Finally, with these anticipated physiological changes, we also expected that perceived exertion and muscle pain would increase during cycling with BFR.

## Materials and Methods

### Participants

Ten active men between 18 and 44 years volunteered to participate in this study (Table 1). All participants self-reported that they performed aerobic exercise at moderate to high-intensity for at least 150 min/week, which is consistent with ACSM guidelines (Garber et al., 2011). Body composition and lower limb lean mass were assessed using dual-energy X-ray absorptiometry (Discovery Wi, Hologic Inc., Marlborough, MA, United States). Participants were excluded from the study if they used nicotine products, had diabetes, or had any cardiopulmonary disorders. Based on pilot data, *a priori* power analysis revealed a sample of 10 participants was adequate to detect a change in blood flow between the BFR conditions (effect size *d* = 0.57) given an alpha of 0.05, with a power of 0.8. Following the initial screening, participants were informed of the purpose of the study, the risks involved, and gave informed written consent. This study was approved by the Institutional Review Board at Michigan Technological University.

**Table 1:** Participant characteristics.

Height (cm)	179 ± 6
Body mass (kg)	79 ± 8
Age (year)	26 ± 6
Body fat (%)	18 ± 4
Lower limb lean mass (kg)	21 ± 2
VO_2peak_ (ml/kg/min)	53 ± 6
Heart rate peak (b/min)	191 ± 9
Limb occlusion pressure (mmHg)	208 ± 19

Values are expressed as mean ± SD. LLLM, Lower limb lean mass.

### Study Overview

**Figure 1:**
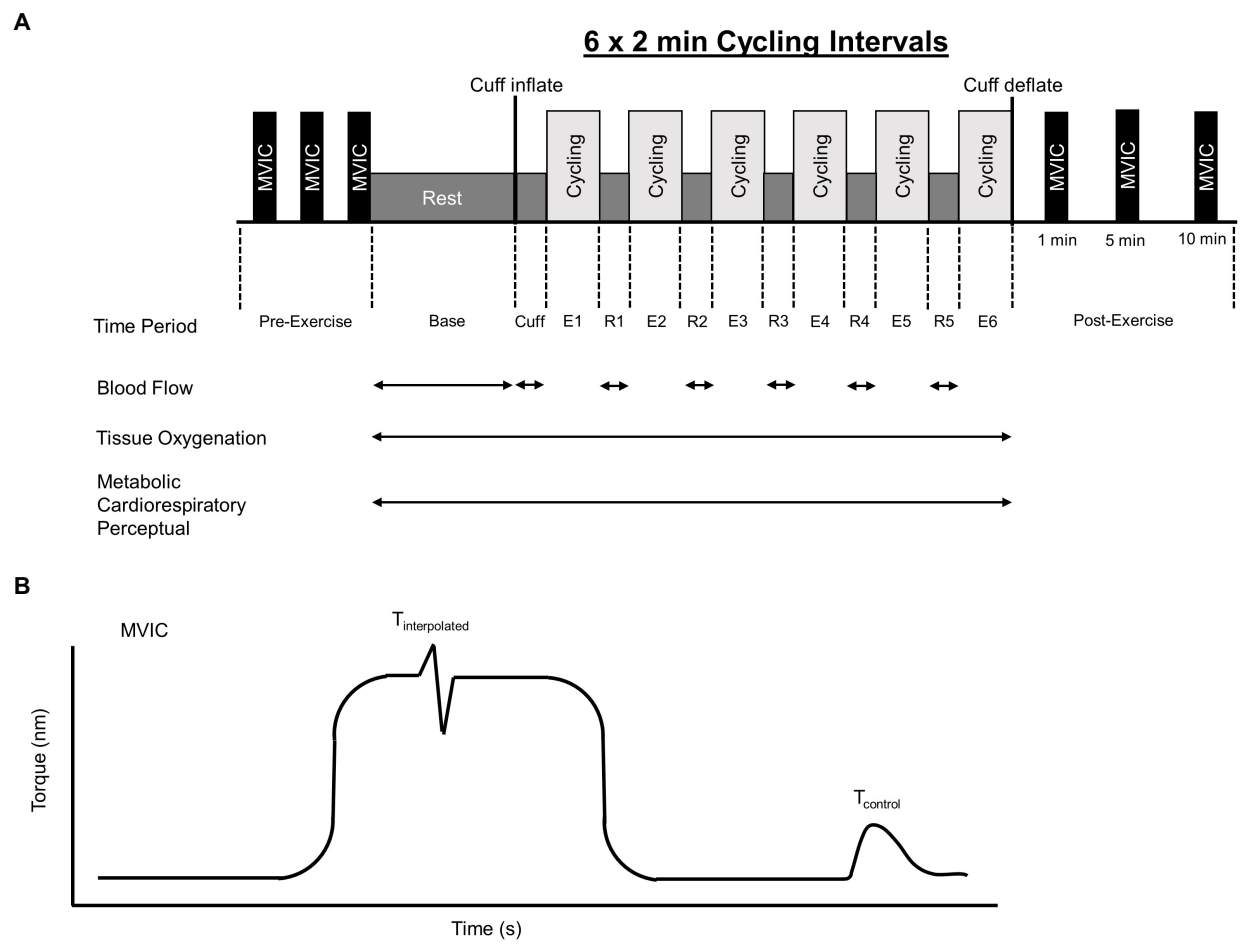
Overview of intermittent cycling protocol which consisted of 6 × 2 min intervals **(A)**. Base, Cuff, E, and R denote baseline, cuff inflate, exercise interval, and recovery interval periods, respectively. Blood flow, tissue oxygenation, metabolic, cardiorespiratory, and perceptual responses were measured during the cycling protocol. Maximal knee extensor voluntary isometric torque (MVIC) was assessed before and 1, 5, and 10 min after exercise. Knee extensor MVIC torque-time profile **(B)**. Voluntary activation was assessed using the interpolated twitch technique and calculated as voluntary activation = 100 × (1 − *T*_interpolated_/*T*_control_), where *T*_interpolated_ was the size of the interpolated twitch and *T*_control_ was the amplitude of the control twitch produced by stimulation of the muscle in a relaxed but potentiated state.

In this investigation, we used a single group repeated measures design. Participants reported to the laboratory on five separate days separated by at least 48 h. Participants were instructed to avoid vigorous physical activity for 24 h prior to each session. All laboratory visits were performed at approximately the same time of day, in a thermoneutral environment. During the initial laboratory visit, participants were familiarized with the measurement of neuromuscular function, performed a submaximal cycling protocol, and completed a graded exercise test for the determination of peak oxygen consumption (VO_2peak_). For the remaining experimental laboratory visits, participants completed one of the four cycling conditions in a randomized order (i.e., simple randomization). The conditions consisted of: (1) low-load cycling at 40% VO_2peak_ without BFR (LL), (2) low-load cycling at 40% VO_2peak_ with BFR set at 60% limb occlusion pressure (BFR60), (3) low-load cycling at 40% VO_2peak_ with BFR set at 80% occlusion pressure (BFR80), and (4) high-load cycling at 80% VO_2peak_ without BFR (HL). These conditions were chosen as they are representative of current guidelines for aerobic exercise with and without BFR (Garber et al., 2011; Patterson et al., 2019) and are similar to previous investigations which have been shown to improve muscular function and aerobic capacity following cycling training with BFR (Abe et al., 2010; Corvino et al., 2014; De Oliveira et al., 2016). Prior to each cycling trial, baseline neuromuscular function was assessed, and limb occlusion pressure was identified. Participants then completed an intermittent cycling protocol (six sets of 2 min cycling intervals with 1 min recovery between sets). Tissue oxygenation and gas exchange data were recorded throughout the cycling trial. Blood flow was measured during the recovery periods. Immediately after the cycling trial, neuromuscular function was assessed again (Figure 1).

### Oxygen Consumption and Peak Aerobic Power

To establish a linear relationship between steady-state VO_2_ and power output, participants completed a submaximal cycling protocol on an electromagnetically braked cycle ergometer (Velotron Elite; RacerMate Inc., Seattle, WA, United States). Specifically, participants cycled at 40, 80, 120, and 160 W for 4 min using a self-selected pedaling rate. Gas exchange data were measured continuously using open-circuit spirometry (True Max 2,400; Parvo Medics, Sandy, UT, United States). The metabolic measurement system was calibrated with a 3 L calibration syringe and medical gases of known concentrations (16.00% O_2_, 4.00% CO_2_, and balanced N_2_). Heart rate was measured continuously using a Polar transmitter (Polar Electro OY, Kempele, Finland). Gas exchange and heart data were averaged every 15 s throughout the test. After a 15 min break, participants then performed a graded exercise test until task failure as described by Lucia et al. (1999). The protocol began at 40 W and increased 5 W every 12 s. The test was terminated voluntarily by the participant, or when pedaling rate could no longer be maintained above 70 rpm, despite verbal encouragement. The highest 30 s average of VO_2_ and heart rate achieved during the test were recorded. Power outputs that elicited 40 and 80% of VO_2peak_ were estimated by interpolating the linear relationship between VO_2_ and power output.

### Cycling Exercise

Prior to the intermittent cycling protocol, participants rested on the cycle ergometer for 5 min for collection of baseline responses. Following this baseline period, the cuff was inflated to 60 or 80% limb occlusion pressure (BFR conditions only) while participants rested for 1 min. Note that, for the LL and HL cycling conditions, 1 min of rest was also provided. Subsequently, participants completed six sets of 2 min cycling intervals with 1 min between sets. An intermittent cycling protocol was selected because previous research suggests that work-rest periods while keeping the pressure cuff inflated has benefits over continuous cycling with BFR (Corvino et al., 2017). For the BFR cycling conditions, blood flow was restricted in each leg using a 10 cm wide nylon pneumatic cuff (Hokanson, Belleview, WA, United States) wrapped around the thigh at the most proximal location. The cuff pressure was set and maintained using a rapid cuff inflator (Hokanson, Belleview, WA, United States). This system is commonly used for administering BFR and its important to acknowledge that pressure set on the system can differ slightly from the actual cuff-to-limb interface pressure (Hughes et al., 2018b). The pressure in the cuff was sustained throughout the entire cycling protocol and deflated immediately after the participant completed the last set of cycling.

### Blood Flow

Blood flow was measured in the superficial femoral artery just distal to the pressure cuff. Blood velocity (*V*_mean_) and vessel diameter (*V*_d_) were measured with a Logiq *E* ultrasound system (General Electric Medical Systems, Milwaukee, WI, United States) equipped with a linear array transducer operating at an imaging frequency of 12 MHz and Doppler frequency of 5 MHz. Doppler pulse wave spectrum and ultrasound images were continuously recorded throughout each time period. Vessel diameters were determined by averaging the perpendicular distance between the superficial and deep walls of the superficial femoral artery at three nonconsecutive R waves during the last 15 s of each recording. Measurements of *V*_mean_ were obtained with the probe positioned to maintain an insonation angle of ≤60°. Mean blood velocity was averaged across 15 s intervals throughout the recording. Importantly, blood velocity data obtained with Doppler ultrasound are reliable (Nyberg et al., 2018) which is notable given the complex nature of blood velocity during dynamic muscle contractions. Using arterial diameter (*V_d_*) and mean blood velocity (*V*_mean_), blood flow was calculated as $\mathrm{Blood flow}=V_{\mathrm{mean}}\times \pi \times \frac{V_{d}}{2}\times 60\;$ as described by Wray et al. (2011). Blood flow was averaged throughout the 1 min recovery interval.

Limb occlusion pressure was determined while the participant was seated on the cycle ergometer. Their right foot was positioned on a stool next to the ergometer. Their hip was abducted slightly and their knee angle was ~90°. The pressure cuff was wrapped around the right thigh, at the same position as exercise. The ultrasound probe was positioned distal to the cuff over the superficial femoral artery. Limb occlusion pressure was identified by inflating the cuff to 75 mmHg, and slowly increasing the pressure until blood velocity reached zero based on the absence of the Doppler spectrum. The minimum pressure required to do this was recorded as the limb occlusion pressure. The measurement of limb occlusion pressure using this method in our laboratory was reliable across exercise sessions (ICC = 0.89; 95% CI 0.80–0.94).

### Tissue Oxygenation

A continuous-wave near-infrared spectroscopy device (PortaLite; Artinis Medical Systems BV, Elst, Netherlands) was utilized to detect changes in the concentrations of oxygenated hemoglobin and deoxygenated hemoglobin. Wavelengths (760 and 850 nm) were emitted from LEDs with an inter-optode distance of 3.5 cm. A differential path-length factor of 4.0 was used to correct for photon scattering within the tissue. Data were collected at 10 Hz (Oxysoft; ArtinisMedical Systems BV, Elst, Netherlands). The sensor was placed midway between the anterior superior iliac spine and the proximal patella parallel to the muscle fibers. The sensor was attached with double-sided tape and wrapped in an opaque bandage to prevent ambient light from reaching the sensor.

$\mathrm{Tissue Saturation Index}=\frac{\mathrm{oxyhemoglobin}}{\mathrm{deoxyhemoglobin}+\mathrm{oxyhemoglobin}}$ was calculated using integrated software (Oxysoft; ArtinisMedical Systems BV, Elst, Netherlands). The average tissue saturation index over the last 10 s of each time period was recorded. Changes in deoxyhemoglobin were assessed by calculating the difference between the average value of the last 10 s of each time period and the last 10 s of data recorded prior to inflating the cuff. This near-infrared spectroscopy system is reliable for measurement of tissue saturation index during leg exercise (Lucero et al., 2018).

### Metabolic, Cardiorespiratory, and Perceptual Responses

Oxygen consumption, ventilation, and heart rate were recorded using the metabolic measurement system described above. These data were averaged over the last 30 s of the exercise and recovery time periods. Perceptual responses (rating of perceived exertion and pain) were recorded during the last 30 s of each exercise and recovery period. Whole body rating of perceived exertion was assessed using a Borg 6–20 scale (Borg, 1982). Pain was assessed using an 11-point numeric rating scale (DeLoach et al., 1998). A blood sample was collected from the fingertip (5 μl) during baseline and 1 min after the final exercise interval from which blood lactate concentration was determined (Lactate Plus; Nova Biomedical, Waltham, MA, United States). This device provides a valid and reliable measure of blood lactate concentration (Hart et al., 2013).

### Neuromuscular Function

Participants were positioned on an isokinetic dynamometer (Biodex 4, Biodex Medical Systems, NY, United States) at a hip angle of 85° and a knee angle of 90°. A seat belt and ankle strap were used to minimize hip and ankle movement. To measure knee extension maximal voluntary isometric contraction (MVIC) torque, participants were instructed to “push as hard and as fast as possible” against an immoveable pad. Standardized verbal encouragement was provided to the participant. Evoked torque was elicited by transcutaneous electrical stimulation over the knee extensors using a computer-controlled stimulator (D185; Digitimer, Welwyn Garden City, United Kingdom). The stimulating cathode was placed over the quadriceps femoris 10 cm distal to the anterior superior iliac spine and the anode was placed 2 cm proximal to the proximal border of the patella (Pietrosimone et al., 2011). An electrical pulse (singlet, square wave, and 100 μs duration) was used to elicit a superimposed twitch at the peak torque level during the MVIC, and an additional potentiated resting twitch was triggered upon relaxation (~2 s) following the MVIC. Stimulation intensity was determined based off no increase in twitch force despite increasing the stimulation current. A further increase of 20% was added to ensure that the stimulation was supramaximal. Voluntary activation was assessed using the interpolated twitch technique and calculated as $\mathrm{Voluntary activation}=100\times \left(1-\frac{T_{\mathrm{interpolated}}}{T_{\mathrm{control}}}\right)$ where *T*_interpolated_ was the size of the interpolated twitch and *T*_control_ was the amplitude of the control twitch produced by stimulation of the muscle in a relaxed but potentiated state (Gandevia, 2001). Rate of torque development, time to peak torque, as well as the time to half-relaxation were calculated using a customized routine (Spike 2; Cambridge Electronics Design, Cambridge, United Kingdom) described by Yoon et al. (2007). At the start of the MVIC, rate of torque development was calculated as the peak tangential torque using a moving mean method of the torque-time curve over the first 400 ms from the onset of contraction. Time to peak torque was defined as the slope of the force-time curve from baseline to peak *T*_control_ torque. Half-relaxation time is defined as the slope of the line from peak *T*_control_ torque to half its value. These measurements were obtained three times at baseline with 2 min rest between contractions the highest of which was used for analysis. Post-exercise measurements were then recorded from a single contraction at 1, 5, and 10 min after the exercise. The measurement of MVIC torque using this equipment in our laboratory was reliable across exercise sessions (ICC = 0.91; 95% CI 0.76–0.98).

### Statistical Analysis

Separate two-way repeated measures analysis of variance (ANOVA) procedures were used to evaluate the effect of cycling condition and time (baseline, cuff inflate, and recovery interval number) on changes in blood flow. If a significant main effect of cycling condition was found, then subsequent *post-hoc* tests (Fisher’s least significant difference) were used to determine where the differences occurred. If a significant effect of time was found, blood flow values over the recovery intervals were pooled together and additional paired samples *t*-tests were performed to analyze simple main effects.

Separate two-way repeated measures ANOVA procedures were used to evaluate the effect of cycling condition and time (baseline, cuff inflate, exercise interval number, and recovery interval number) on changes in tissue saturation index, deoxyhemoglobin, VO_2_, heart rate, ventilation, RPE, and pain. If a significant main effect of cycling condition was found, then subsequent *post-hoc* tests (Fisher’s least significant difference) were used to determine where the differences occurred. Additionally, if a significant main effect of time or condition was found for tissue saturation index or deoxyhemoglobin, data for exercise intervals and also recovery intervals were pooled and additional paired samples *t*-tests were used to evaluate simple main effects.

A repeated measures ANOVA was used to evaluate the interaction of cycling condition and time (baseline to post-exercise) on whole blood lactate. If a significant interaction was identified, then a series of 2 × 2 repeated measures ANOVAs comparing each cycling condition at baseline and post-exercise were used to determine where the interactions occurred.

Finally, two-way repeated measures ANOVAs were used to evaluate the effect of cycling condition and time (baseline, post-1 min, post-5 min, and post-10 min), on changes in MVIC, *T*_control_, voluntary activation, rate of torque development, time to peak torque, and half-relaxation time. If a significant main effect of cycling condition was found, then subsequent *post-hoc* tests (Fisher’s least significant difference) were used to determine where the differences occurred. If a significant main effect of time was found paired samples *t*-tests were used to evaluate simple effects of time. If a significant interaction of cycling condition and time was found, a series of 2 × 2 repeated measures ANOVAs comparing each cycling condition at baseline and 1 min post-exercise were used to determine where the interactions occurred. Partial eta squared (*η_p_*^2^) was calculated as a measure of effect sizes with *η_p_*^2^ ≥ 0.01 indicating small, ≥0.059 medium, and ≥0.138 large effects, respectively (Cohen, 1988). Statistical procedures were performed using SPSS 22 (Armonk, NY, United States). Data are reported as mean ± SD and alpha was set to 0.05.

## Results

### Cycling Trials

Cuff pressures for the BFR60 and BFR80 cycling conditions were 125 ± 12 and 164 ± 15 mmHg, respectively. Mean power outputs were 89 ± 18 W for the LL, BFR60, and BFR80 cycling conditions and 240 ± 36 W for the HL cycling condition. Blood flow at baseline did not differ between conditions (*p* = 0.45). Likewise, LOP did not differ between the BFR60 and BFR80 cycling conditions (207 ± 21 vs. 205 ± 19 mmHg, *p* = 0.46). Two participants were unable to complete the last cycling interval in the BFR80 condition due to lightheadedness and/or intense pain at the site of the cuff. The cuff pressure was therefore released, and these side-effects were greatly reduced. These participants still completed the post-exercise assessment of neuromuscular function 1 min following the release of the occlusion cuff. For these two participants, tissue oxygenation, metabolic, cardiorespiratory, and perceptual data during the final interval were substituted using mean substitution.

### Blood Flow

**Figure 2:**
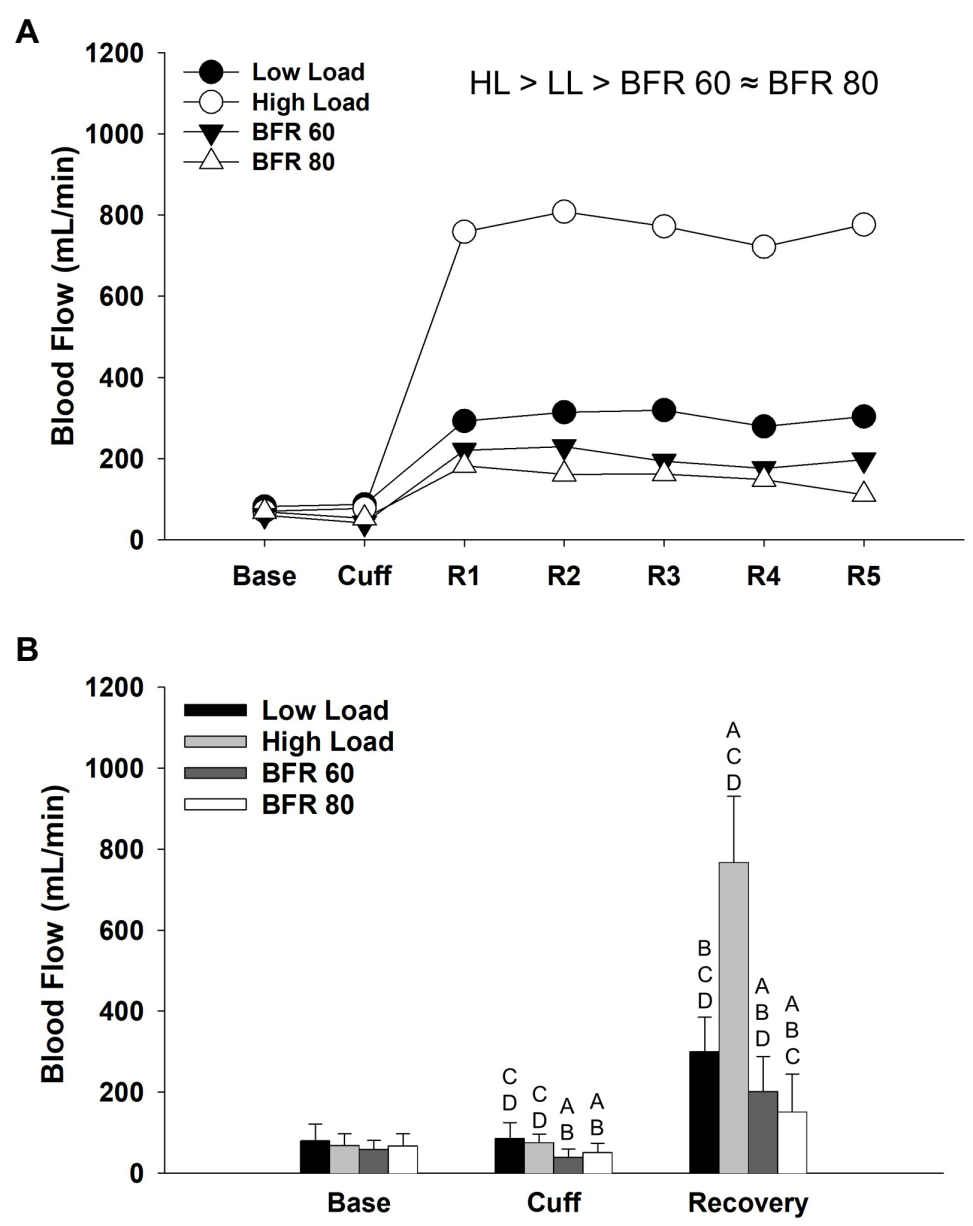
Time course of alterations in superficial femoral artery blood flow across the different cycling conditions **(A)**. Significant main effect of condition is indicated above figure, ≈ ( *p* > 0.05), < or > ( *p* < 0.05). Base, Cuff, and R denote baseline, cuff inflate, and recovery interval periods, respectively. Data are reported as means and standard deviation bars were removed for clarity. Mean superficial femoral artery blood flow during baseline, cuff inflate, and recovery periods **(B)**. Blood flow different from LL, HL, BFR60, and BFR80 ( *p* < 0.05) are indicated by ^A^, ^B^, ^C^, and ^D^, respectively. Data are reported as mean ± SD.

The repeated measures ANOVA revealed significant main effects of cycling condition (*p* < 0.01, *η_p_*^2^ = 0.887) and time (*p* < 0.01, *η_p_*^2^ = 0.933) and cycling condition × time interaction (*p* < 0.01, *η_p_*^2^ = 0.802) on blood flow. In general, blood flow was reduced in the BFR cycling conditions compared to the non-BFR conditions (Figure 2A) and tended to decrease further in the BFR80 condition compared to BFR60 (*p* = 0.07). Within in each time period, blood flow did not differ between conditions at baseline, decreased with cuff inflation for BFR60 and BFR80 (both *p* < 0.05), and differed between all conditions during the recovery periods (HL > LL > BFR60 > BFR80; all *p* < 0.05, Figure 2B).

### Tissue Oxygenation

**Figure 3:**
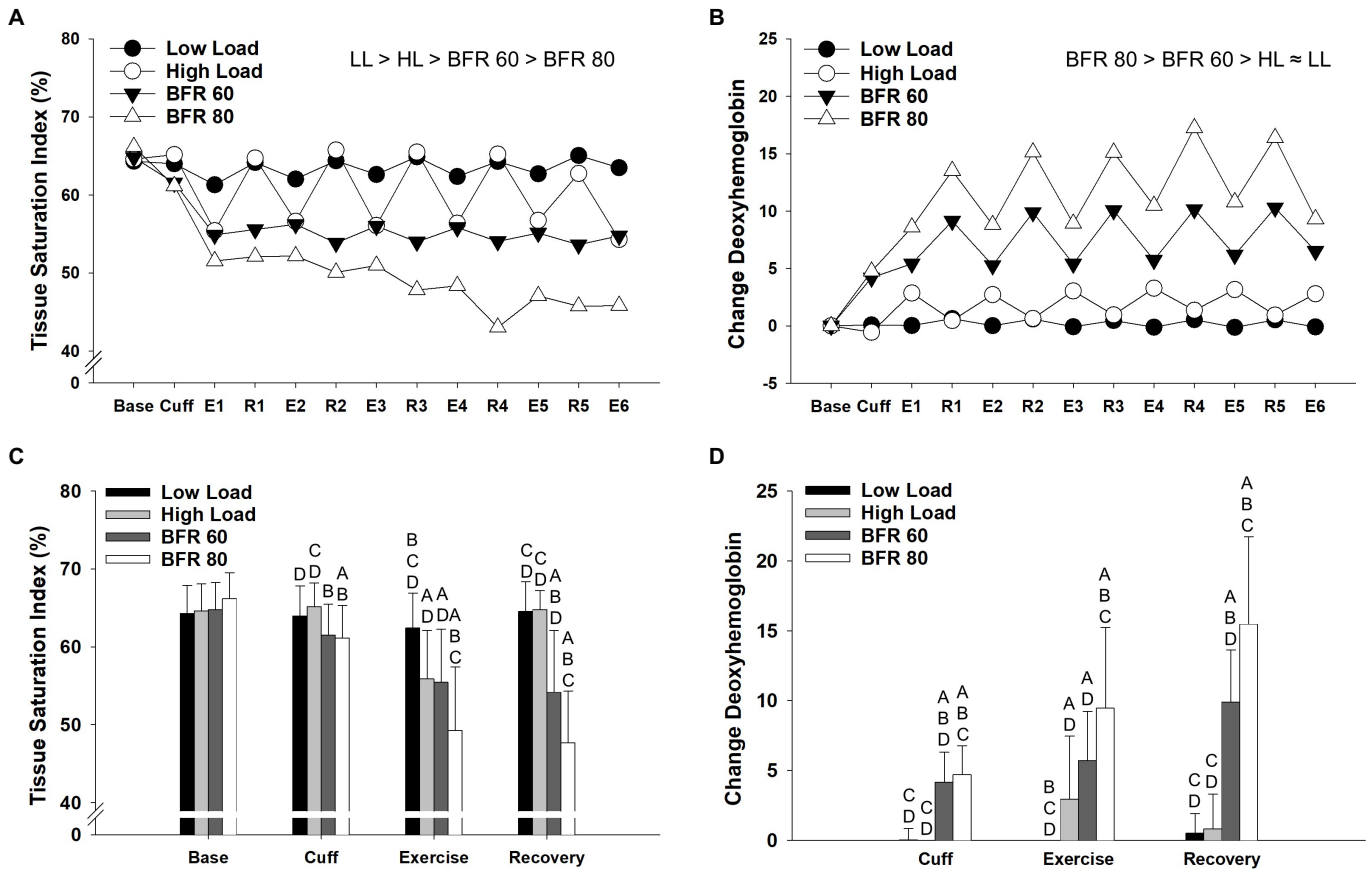
Time course of alterations in tissue saturation index **(A)** and concentrations of deoxyhemoglobin **(B)** across the different cycling conditions. Significant main effects of condition are indicated above individual figures, ≈ ( *p* > 0.05), < or > ( *p* < 0.05). Base, Cuff, E, and R denote baseline, cuff inflate, exercise interval, and recovery interval periods, respectively. Data reported as means and standard deviation bars were removed for clarity. Mean values for tissue saturation index **(C)** and concentrations of deoxyemoglobin **(D)** during baseline, cuff inflate, exercise, and recovery periods. Tissue saturation index and concentrations of deoxyhemoglobin different from LL, HL, BFR60, and BFR80 ( *p* < 0.05) are indicated by ^A^, ^B^, ^C^, and ^D^, respectively. Data are reported as mean ± SD.

Results from the repeated measures ANOVA procedures for tissue saturation index and deoxyhemoglobin revealed significant main effects of cycling condition (*p* < 0.01, *η_p_*^2^ = 0.741; *p* < 0.01, *η_p_*^2^ = 0.783, respectively) and time (*p* < 0.01, *η_p_*^2^ = 0.599; *p* < 0.01, *η_p_*^2^ = 0.687, respectively) as well as a cycling condition × time interaction (*p* < 0.01, *η_p_*^2^ = 0.511; *p* < 0.01, *η_p_*^2^ = 0.683, respectively). Overall, tissue saturation index was higher in LL, and HL, than it was in the BFR conditions (all *p* < 0.05, Figure 3A). Tissue saturation index was reduced in the BFR80 condition compared to the BFR60 (*p* < 0.01). Changes in tissue saturation index within each time period are illustrated in Figure 3C. Compared to exercise, tissue saturation index increased during recovery for HL and LL cycling conditions (both *p* < 0.05). There was no difference in tissue saturation index during exercise and recovery within the BFR60 and BFR80 conditions. In general, concentrations of deoxyhemoglobin were higher in the BFR conditions (all *p* < 0.05) and increased with pressure (*p* < 0.05, Figure 3B). There was a trend for concentrations of deoxyhemoglobin to be lower in LL than HL (*p* = 0.06). Changes in concentrations of deoxyhemoglobin with in time period are illustrated in Figure 3D. Deoxyhemoglobin did not differ between exercise and recovery periods in the HL and LL conditions (both *p* > 0.05) but increased during the recovery period for the BFR60 and BFR80 conditions (both *p* < 0.01).

### Metabolic, Cardiorespiratory, and Perceptual Responses

**Figure 4:**
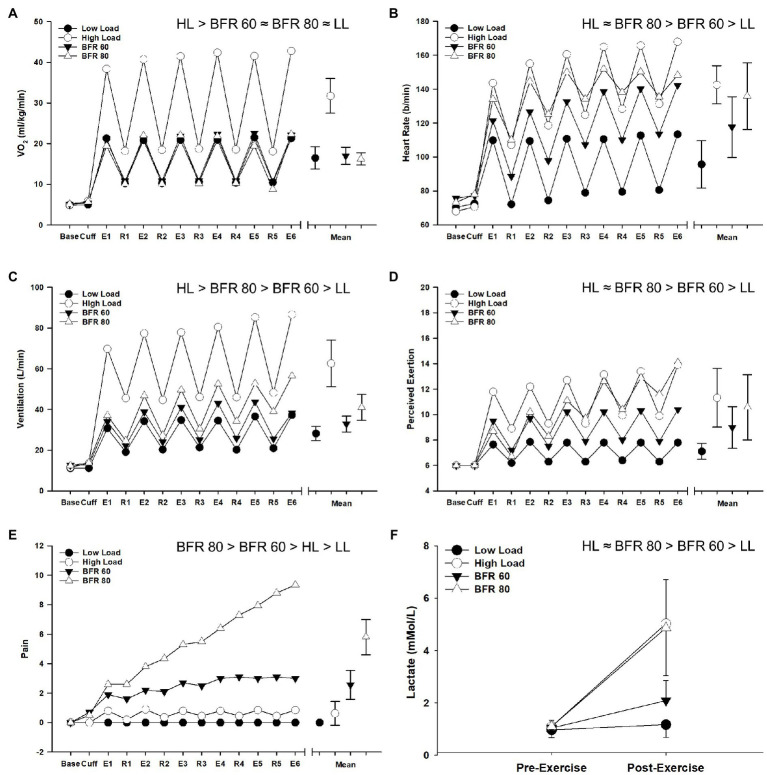
Time course of alterations in VO_2_ **(A)**, heart rate **(B)**, ventilation **(C)**, RPE **(D)**, pain **(E)**, and blood lactate **(F)** across the different cycling conditions. Significant main effects of condition are indicated above individual figures, ≈ ( *p* > 0.05), < or > ( *p* < 0.05). Base, Cuff, E, and R denote baseline, cuff inflate, exercise interval, and recovery interval periods, respectively. Data are reported as means and standard deviation bars were removed for clarity. Note that, for Panels **(A–E)**, a collapsed mean for the exercise interval and recovery periods is illustrated to the right (mean ± SD).

The repeated measures ANOVA procedures revealed main effects of cycling condition (all *p* < 0.01, all *η_p_*^2^ > 0.678) and time (all *p* < 0.01, all *η_p_*^2^ > 0.818) as well as a cycling condition × time interaction (all *p* < 0.01, all *η_p_*^2^ > 0.511) for all variables (VO_2,_ heart rate, ventilation, RPE, pain, and lactate). Due to the intermittent nature of the cycling protocol, VO_2_ displayed a general “sawtooth” pattern for all conditions and overall was highest for HL (all *p* > 0.05, Figure 4). Heart rate was elevated in the BFR conditions compared to LL (both *p* < 0.05) but heart rate in the BFR80 condition did not significantly differ from HL (*p* = 0.30). Ventilation was highest in the HL condition followed by BFR80, BFR60, and LL, respectively (all *p* < 0.05). Cycling with BFR caused an increase in RPE compared to LL (both *p* < 0.05), but RPE in the BFR80 condition was not significantly different from HL (*p* = 0.30). Pain was generally low during LL and HL but increased with BFR and increased pressure (all *p* < 0.05). Most notably, pain during BFR80 increased steadily and approached maximum values at the end of the final interval. Compared to baseline, end exercise whole blood lactate increased in the HL, BFR60, and BFR80 conditions (all *p* < 0.05). The increase in blood lactate for HL and BFR80 was greater than that for BFR60 but did not significantly differ between HL and BFR80 conditions (*p* = 0.86).

### Neuromuscular Function

**Figure 5:**
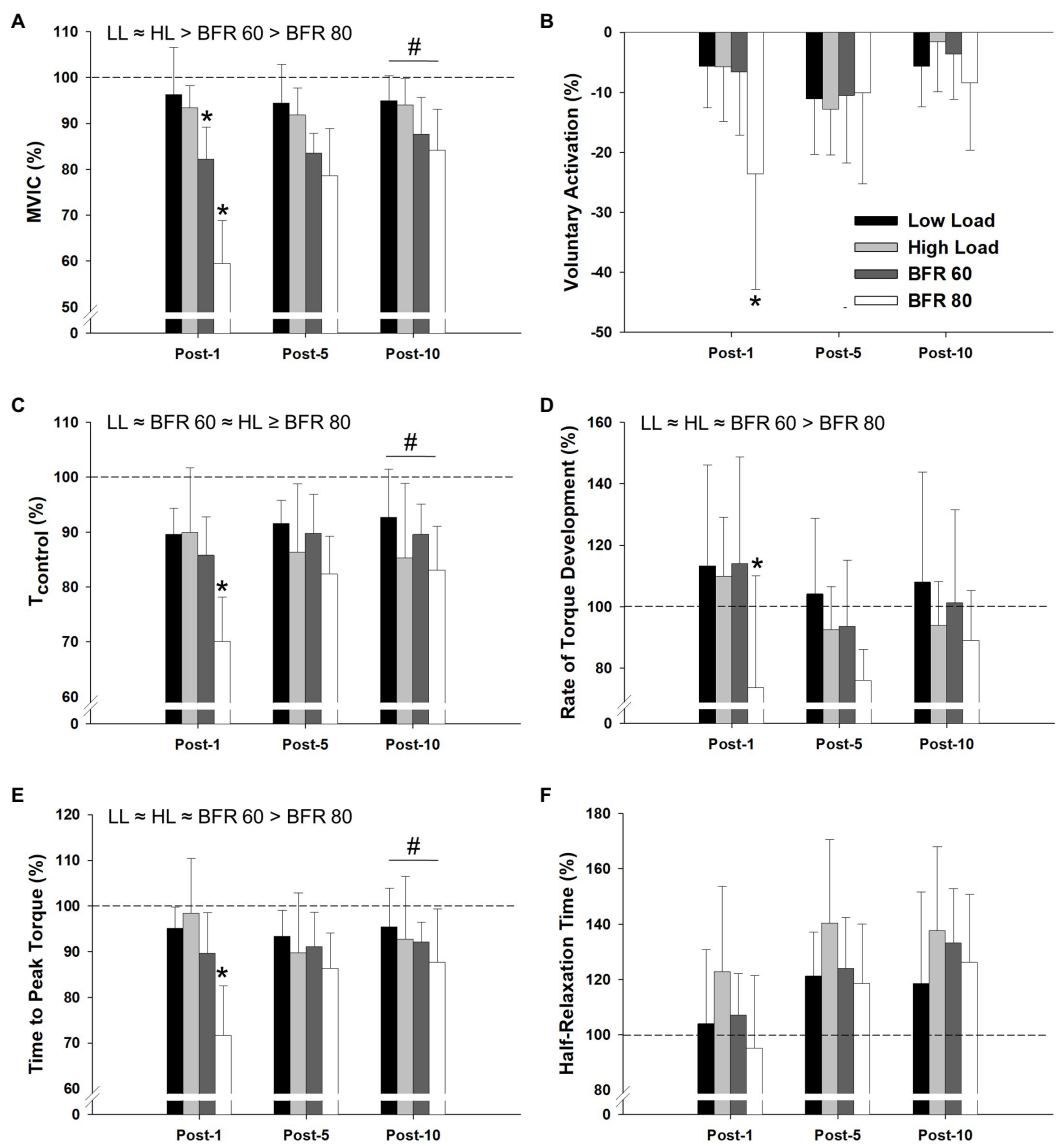
Relative changes in maximal voluntary isometric contraction torque (MVIC), voluntary activation, evoked twitch torque (*T*_control_), rate of torque development, time to peak torque, and half-relaxation time, as assessed at 1, 5, and 10 min post-exercise, are illustrated in panel **(A–F)**, respectively. Dashed line indicates baseline pre-exercise value. Significant main effects of condition are indicated above individual figures, ≈ (denotes *p* > 0.05), < or > (denotes *p* < 0.05). Note that, *T*_control_ ≥ indicated BFR80 is different than LL and BFR60, but not different than HL. Condition × time interaction (baseline to 1 min post-exercise) different than all other conditions (^*^p < 0.05). All conditions different from baseline at post-10 min (^#^*p* < 0.05). Data are reported as mean ± SD.

The repeated measure ANOVA procedures indicated significant main effects of cycling condition for MVIC torque, *T*_control_, rate of torque development, and time to peak torque (all *p* < 0.01, all *η_p_*^2^ > 0.348; Figure 5). Significant main effects of time were found for MVIC, voluntary activation, *T*_control_, Time to peak torque, and half-relaxation time (all *p* < 0.01, all *η_p_*^2^ > 0.525). Significant cycling condition × time interactions were found for MVIC, voluntary activation, *T*_control_, time to peak torque, and rate of torque development (*p* < 0.01, all *η_p_*^2^ > 0.258). In general, MVIC torque produced in the BFR conditions was lower than the non-BFR conditions and was further reduced with increased pressure. Reductions in MVIC torque from baseline to 1 min post-exercise for the BFR60 condition were greater than those for LL and HL (both *p* < 0.05) but less than those for BFR80 (*p* = 0.02). Compared to baseline, MVIC torque was reduced at 10 min post-exercise for all conditions and was lowest for the BFR80 condition (all *p* < 0.05). Reductions in voluntary activation from baseline to 1 min post-exercise were greater in BFR80 compared to all other conditions (all *p* < 0.05). Voluntary activation did not differ from baseline at 10 min post in any condition (all *p* > 0.05). For *T*_control_, BFR80 was lower than LL and BFR60 (both *p* < 0.05), but not different than HL (*p* = 0.10). The baseline to 1 min post-exercise reduction in *T*_control_ was greater in BFR80 than all other conditions (all *p* < 0.05). The *T*_control_ for all of the conditions were reduced 10 min post-exercise compared to baseline (all *p* < 0.05). BFR80 was generally lower than all other conditions for rate of torque development and time to peak torque (all *p* < 0.05). Similarly, the baseline to 1 min change in rate of torque development and time to peak torque for BFR80 was greater than all other conditions (all *p* < 0.05). The rate of torque development for both BFR conditions was reduced 10 min post-exercise (both *p* < 0.05). Compared to baseline, time to peak torque was reduced for all conditions 10 min post-exercise (all *p* < 0.05). Finally, half-relaxation time was not significantly different than baseline 10 min post-exercise in the LL condition (*p* = 0.13) but was increased in all other conditions (all *p* < 0.05).

## Discussion

In this investigation, we integrated measurements of blood flow, tissue oxygenation, and neuromuscular function to better characterize the acute effects of BFR during aerobic exercise. Our main findings were that cycling with BFR (1) caused a reduction in blood flow and tissue oxygenation an augmented metabolite accumulation compared to LL cycling, (2) resulted in lower cardiorespiratory responses compared to HL cycling, and (3) compromised knee extensor torque primarily through peripheral mechanisms. Collectively, these results indicate that both BFR60 and BFR80 provided considerable metabolic stress and resulted in neuromuscular fatigue. While increasing the cuff pressure increased metabolic stress and neuromuscular fatigue, it also increased pain and thus high pressures may not be feasible.

### Blood Flow and Tissue Oxygenation

To the best of our knowledge, we are the first group to report changes in blood flow (as assessed in the superficial femoral artery) following aerobic exercise with BFR. Specifically, compared to the LL condition, blood flow during the intermittent recovery period was reduced by ~33% and ~50% in the BFR60 and BFR80 conditions, respectively. These results from aerobic exercise with BFR generally agree with previous literature analyzing reductions in blood flow following resistance exercise with BFR (~30–40%; Takano et al., 2005; Downs et al., 2014; Kilgas et al., 2019; Singer et al., 2020). This reduction in blood flow to the muscle was generally supported by our tissue oxygenation data. Specifically, tissue saturation index was reduced by ~5% and ~15% in the BFR60 and BFR80 conditions. These reductions support previous reports on muscle oxygenation during cycling with BFR. Specifically, Corvino et al. (2017) reported ~6% reduction in tissue saturation index during intermittent cycling with BFR with a cuff pressure of 20 mmHg above occlusion pressure. Additionally, Willis and coworkers (Willis et al., 2018) reported that tissue saturation index was lower than control at 60% of occlusion pressure, but not 45% of occlusion pressure following repeated cycling sprints until exhaustion. Previous authors have hypothesized that lower oxygen availability and venous occlusion induces training adaptions through a combination of cell swelling (Loenneke et al., 2012a), metabolite accumulation (Scott et al., 2014; Cayot et al., 2016), and type two muscle fiber recruitment (Yasuda et al., 2009). Understanding the acute changes in blood flow and tissue saturation during cycling with BFR is key to optimizing the training stimulus. Our data suggest that both BFR60 and BFR80 may serve as appropriate training pressure during cycling with BFR.

### Metabolic, Cardiorespiratory, and Perceptual Responses

Oxygen consumption did not change with the addition of BFR. Previous researchers (Abe et al., 2006; Loenneke et al., 2011) reported an increase in VO_2_ during aerobic exercise with BFR likely due to the metabolic cost of increased heart rate and ventilation. Discrepancies in these findings may be due to the duration of the cycling intervals. Because the intervals in the present study were limited to 2 min, steady-state VO_2_ was not achieved. Heart rate, ventilation, RPE, pain, and lactate all increased with BFR and further increased with higher cuff pressure, which is consistent with previous research (Mendonca et al., 2014; Mattocks et al., 2017). Notably, heart rate and RPE increased in the BFR80 to a level that was not different from HL, additionally muscle pain during the last cycling interval was near maximum values. Therefore, BFR80 may not be tolerable for everyone.

### Neuromuscular Function

Cycling exercise with BFR reduced MVIC torque by ~18% and ~40% in the BFR60 and BFR80 conditions, respectively. These results generally agree with previous reports documenting changes in neuromuscular function following resistance exercise with BFR (Cook et al., 2007; Karabulut et al., 2010; Husmann et al., 2018); however, prior studies examining neuromuscular function following aerobic exercise with BFR have varied (Ogawa et al., 2012; Kim et al., 2015; Willis et al., 2018). These reductions in muscular torque in the current study were likely influenced by peripheral mechanisms as indicated by the reduction in *T*_control_ for both the BFR60 and BFR80 conditions. Exercise with BFR may have caused peripheral fatigue by the reduction in blood flow to the working muscles reducing their energy supply and/or by the accumulation of metabolites inhibiting cross-bridge formation (Husmann et al., 2018).

In addition to peripheral fatigue, central fatigue was also present in the BFR80 condition. Specifically, voluntary activation was 23% lower in the BFR80 condition 1 min after exercise. Willis et al. (2018) reported a 6 and 16% reduction in voluntary activation following a repeated cycling sprint test with BFR at 45 and 60% occlusion pressure to exhaustion. The reduction in voluntary activation in the BFR80 condition could be due to several factors related to exercise with BFR. One explanation includes venous distension, and the accumulation of metabolites, both of which increase the firing rate of nociceptive group IV muscle afferents (Haouzi et al., 1999; Jankowski et al., 2013). As stated above, the BFR80 condition resulted in significant muscle pain, which increased throughout the protocol. Graven-Nielsen et al. (2002) previously reported muscle pain, *via* infusion of hypertonic saline, reduced MVIC torque through a centrally mediated mechanism. Greater neuromuscular fatigue has been associated with increased growth hormone concentrations (Hakkinen and Pakarinen, 1993; Pierce et al., 2006) which may play a role in training adaptations following aerobic and resistance training with BFR. Results from the current study provide evidence of neuromuscular fatigue following aerobic exercise with BFR.

### Cuff Pressure

Proper selection of cuff pressure (e.g., 40–80% of limb occlusion pressure) is important for the safety and effectiveness of exercise with BFR. At rest, application of pressure would initially occlude venous blood flow while maintaining some arterial blood flow until the arterial to venous pressure gradient is eliminated at which arterial blood flow would stop. During exercise, blood flow would increase only if the intramuscular pressure generated during the muscle contraction exceeds the cuff pressure (Kilgas et al., 2019; Singer et al., 2020). By reducing blood flow, this causes a reduction in tissue oxygenation and a build-up of metabolites and fluid within the limb. Previous authors have proposed these as possible mechanisms responsible for the training adaptations following exercise with BFR (Loenneke et al., 2014; Scott et al., 2015). Higher cuff pressures likely result in greater venous occlusion which augments cell swelling by causing blood to pool within the limb, which is supported by data in the present study. Loenneke et al. (2012b) suggested that the ideal cuff pressure necessary for training adaptation is less than previously used in the literature. This could be due to very high cuff pressures further occluding arterial blood flow and thus attenuating the cell swelling response. Increasing cuff pressure also likely increases metabolite accumulation within the limb, which activate the exercise pressor reflex. The activation of the exercise pressor reflex and increased metabolite concentrations may explain the increased heart rate, ventilation, pain, and fatigue seen in the present study. These results may decrease BFR exercise training volume by early termination of the exercise. It should be noted that while BFR80 resulted in a greater increase in metabolite accumulation, it also resulted in substantial pain by the end of the final cycling interval (two participants could not complete the protocol). While both BFR60 and BFR80 reduced tissue oxygenation, increased metabolite accumulation, and caused neuromuscular fatigue, BFR60 was more tolerable.

### Implications

Researchers, clinicians, and practitioners should take great care in selecting cuff pressures as pressure effects acute and likely chronic responses to exercise. In this investigation, cuff pressures of 60 and 80% limb occlusion pressure resulted in reduced blood flow, decreased muscle oxygenation, increased metabolite accumulation, and reduced neuromuscular function, yet only BFR60 resulted in lower metabolic, cardiorespiratory, and perceptual responses than HL cycling. It is important to note that limb occlusion pressures vary by individual, cuff type, cuff width, and body position used (Hughes et al., 2018a; Sieljacks et al., 2018). Therefore, cuff pressures should be based on individual limb occlusion pressure if possible. Using an absolute pressure may result in inconsistent training adaptations or adverse side-effects (Rolnick et al., 2021). Finally, this study highlights cuff pressures that elicit specific stresses to the cardiovascular and neuromuscular systems that may be advantageous for cycling exercise with BFR, which may aid researchers in the development of robust cycling exercise with BFR guidelines (exercise protocols and cuff pressures) for healthy, clinical, and athletic populations.

### Limitations

There are some limitations to our study that must be addressed. First, blood flow measurements using Doppler ultrasound during cycling could not be performed; therefore, we measured blood flow in the superficial femoral artery immediately (within 10 s) after each cycling interval. Although not directly measured, we can speculate that blood flow during exercise was higher than during recovery in the BFR conditions based on the tissue oxygenation data. Additionally, although careful control was taken to minimize day to day variation in blood flow and no differences in baseline blood flow were found, this variation may affect our findings. Second, limb occlusion pressure was measured in the superficial femoral artery at rest while seated on the ergometer. Because limb occlusion pressure is dependent on body position (Hughes et al., 2018a) and deeper arteries will require higher pressures to fully occlude, absolute cuff pressures from the current study may not be applied when limb occlusion pressure is measured in different body position or different arteries. Third, our measurements of tissue oxygenation using near inferred spectroscopy may be affected by adipose tissue, higher skin perfusion during exercise, melanin in the skin, and heterogeneity of blood flow within the muscle (Jones et al., 2016). Finally, our measurements of neuromuscular function were performed 1 min after the conclusion of exercise. This minute was used for the participant to transfer from the cycling ergometer to the isokinetic dynamometer. This time may have masked some of the changes to neuromuscular function following exercise. Moreover, changes in neuromuscular function are task-specific (Bigland-Ritchie et al., 1995), and therefore, caution must be taken when extrapolating these findings to other modes of aerobic exercise with BFR, exercise protocols, and/or other populations including women and older adults.

## Summary

In summary, cycling with BFR decreased blood flow and tissue oxygenation and increased metabolite accumulation when compared to LL cycling without BFR. Moreover, cycling with BFR generally resulted in lower metabolic and cardiorespiratory responses than traditional HL cycling and reduced neuromuscular function primarily by peripheral mechanisms. Additionally, higher cuff pressure not only increased metabolic stress and fatigue but also increased pain. We conclude that intermittent cycling with a cuff pressure of 60% limb occlusion pressure has the potential to strike a balance between reducing blood flow and increasing metabolite stress, which is needed for training adaptations, without causing excessive cardiorespiratory strain and severe pain. Therefore, cycling with this pressure could offer an alternative when high-intensity aerobic exercise is not suitable.

## Data Availability Statement

The raw data supporting the conclusions of this article will be made available by the authors, without undue reservation.

## Ethics Statement

The studies involving human participants were reviewed and approved by the Institutional Review Board at Michigan Technological University. The patients/participants provided their written informed consent to participate in this study.

## Author Contributions

All authors listed have made a substantial, direct, and intellectual contribution to the work, and approved it for publication.

## Funding

Funding to support this investigation was received from the Michigan Space Grant Consortium.

## Conflict of Interest

The authors declare that the research was conducted in the absence of any commercial or financial relationships that could be construed as a potential conflict of interest.

## Publisher’s Note

All claims expressed in this article are solely those of the authors and do not necessarily represent those of their affiliated organizations, or those of the publisher, the editors and the reviewers. Any product that may be evaluated in this article, or claim that may be made by its manufacturer, is not guaranteed or endorsed by the publisher.
